# Unraveling the Interplay Between Metabolism and Neurodevelopment in Health and Disease

**DOI:** 10.1111/cns.70427

**Published:** 2025-05-14

**Authors:** Yanqing He, Kang Xie, Kang Yang, Nianhua Wang, Longbo Zhang

**Affiliations:** ^1^ Department of Neurosurgery, and National Clinical Research Center of Geriatric Disorders Xiangya Hospital, Central South University Changsha China; ^2^ Department of Neurosurgery Xiangya Hospital, Central South University, Jiangxi (National Regional Center for Neurological Diseases) Nanchang China; ^3^ Department of Neurosurgery Changde Hospital, Xiangya School of Medicine, Central South University (The First People's Hospital of Changde City) Changde Hunan China

**Keywords:** autism spectrum disorders, metabolism, mitochondria, neurodevelopment, neurodevelopmental disorders

## Abstract

**Background:**

Neurodevelopment is a multifaceted and tightly regulated process essential for the formation, maturation, and functional specialization of the nervous system. It spans critical stages, including cellular proliferation, differentiation, migration, synaptogenesis, and synaptic pruning, which collectively establish the foundation for cognitive, behavioral, and emotional functions. Metabolism serves as a cornerstone for neurodevelopment, providing the energy and substrates necessary for biosynthesis, signaling, and cellular activities.

**Results:**

Key metabolic pathways, including glycolysis, lipid metabolism, and amino acid metabolism, support processes such as cell proliferation, myelination, and neurotransmitter synthesis. Crucial signaling pathways, such as insulin, mTOR, and AMPK, further regulate neuronal growth, synaptic plasticity, and energy homeostasis. Dysregulation of these metabolic processes is linked to a spectrum of neurodevelopmental disorders, including autism spectrum disorders (ASDs), intellectual disabilities, and epilepsy.

**Conclusions:**

This review investigates the intricate interplay between metabolic processes and neurodevelopment, elucidating the molecular mechanisms that govern brain development and the pathogenesis of neurodevelopmental disorders. Additionally, it highlights potential avenues for the development of innovative strategies aimed at enhancing brain health and function.

## Graphical abstract

Neurodevelopment is orchestrated by precise metabolic regulation. Key metabolic processes—glucose, lipid, and amino acid metabolism—drive cell proliferation, differentiation, synaptogenesis, and neurotransmitter synthesis. These are tightly integrated with signaling pathways like mTOR, AMPK, and insulin/IGF that regulate neuronal growth and synaptic plasticity. Mitochondrial dynamics and the gut‐brain axis further modulate neurodevelopmental outcomes. Disruption of these metabolic systems is linked to neurodevelopmental disorders, including autism spectrum disorders (ASDs), intellectual disabilities, and epilepsy. Understanding these interactions opens pathways for innovative diagnostics and therapies aimed at restoring brain health.

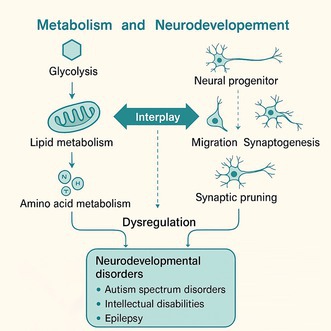

## 1 Introduction

Neurodevelopment is a multifaceted and tightly regulated process that orchestrates the formation, maturation, and functional specialization of the nervous system. It spans embryonic development through early postnatal stages and continues to influence brain plasticity into adulthood. This intricate journey encompasses cellular proliferation, migration, differentiation, synaptogenesis, and synaptic pruning, processes that collectively establish the structural and functional foundations of the nervous system. Understanding neurodevelopment is of paramount importance, as its precise execution underpins cognitive, motor, and sensory functions critical for survival and adaptation.

The significance of neurodevelopment lies in its direct impact on human health and behavior. Proper neurodevelopment not only enables an individual's ability to learn, communicate, and interact with the environment but also sets the stage for lifelong neural plasticity and adaptability. Conversely, disruptions during any phase of neurodevelopment can result in a continuum of neurological and psychiatric disorders. From autism spectrum disorders (ASDs) and intellectual disabilities to epilepsy and schizophrenia, the ramifications of abnormal neurodevelopment extend across a spectrum of conditions, emphasizing the necessity of unraveling the underlying mechanisms.

### 1.1 The Complexity of Neurodevelopmental Processes

The orchestrated progression of neurodevelopment involves a symphony of genetic, epigenetic, and environmental factors. Neural progenitor cells undergo proliferation and differentiation to generate diverse neuronal and glial populations. These cells migrate to their destined locations within the brain and spinal cord, guided by molecular cues and structural scaffolding. Upon reaching their targets, neurons extend axons and dendrites to form synaptic connections, creating neural circuits capable of processing and transmitting information [1]. Synaptic plasticity, the ability of synapses to strengthen or weaken over time, further refines these circuits in response to activity and experience [2].

Adding to this complexity, neurodevelopment operates within a precise temporal framework. Each stage, from neural tube formation to the establishment of cortical layers, requires an intricate interplay between intrinsic genetic programs and extrinsic modulatory signals. Perturbations at any stage can alter developmental trajectories, leading to cascading effects that may not become evident until later in life. For instance, errors in neuronal migration during early development are linked to cortical malformations such as focal cortical dysplasia (FCD) [3], which are often associated with epilepsy.

### 1.2 The Role of Metabolism in Neurodevelopment

Metabolism emerges as a cornerstone in the regulation of neurodevelopmental processes. Beyond providing energy to fuel cellular activities, metabolism contributes substrates for biosynthetic pathways and influences signaling cascades critical for development. During early embryogenesis, the metabolic landscape shifts dynamically to meet the changing demands of proliferating and differentiating neural cells. Key metabolic pathways, such as glycolysis, oxidative phosphorylation (OXPHOS), and the pentose phosphate pathway (PPP), support the biosynthesis of nucleotides, lipids, and proteins essential for cellular growth and division [4, 5]. Mitochondria, often referred to as the powerhouses of the cell, play a pivotal role in neurodevelopment. These organelles regulate adenosine triphosphate (ATP) production, calcium homeostasis, and the generation of reactive oxygen species (ROS), all of which are crucial for cellular signaling and survival. Emerging evidence suggests that mitochondrial dynamics, including fission and fusion, are tightly linked to neuronal differentiation and synaptic function [6]. Disruptions in mitochondrial metabolism are increasingly recognized as contributors to neurodevelopmental disorders, highlighting the organelle's central role in brain health [7].

Another critical aspect of neurodevelopmental metabolism is the involvement of metabolic signaling pathways, such as the mammalian target of rapamycin (mTOR) pathway. mTOR integrates signals from nutrients, growth factors, and cellular energy status to regulate processes like protein synthesis, autophagy, and cytoskeletal dynamics. Aberrant mTOR signaling has been implicated in a range of neurodevelopmental disorders, including tuberous sclerosis complex (TSC) and FCD, underscoring the pathway's importance in maintaining developmental homeostasis [8, 9].

### 1.3 Metabolic Dysregulation and Neurodevelopmental Disorders

The intricate relationship between metabolism and neurodevelopment implies that even subtle metabolic dysregulation can have profound consequences. Dysregulated energy metabolism, oxidative stress, and impaired biosynthetic capacity can disrupt the tightly controlled processes of neural proliferation, migration, and synaptogenesis. For instance, defects in mitochondrial function have been implicated in conditions such as Leigh syndrome and other mitochondrial encephalopathies, which often present with developmental delays, seizures, and cognitive impairments [7]. Abnormalities in glucose metabolism also play a critical role in neurodevelopmental disorders. The brain's reliance on glucose as its primary energy source makes it particularly vulnerable to disruptions in glucose transport and utilization. Disorders such as glucose transporter 1 (GLUT1) deficiency syndrome highlight the consequences of impaired glucose transport across the blood–brain barrier (BBB), leading to developmental delay and epileptic encephalopathy [5]. Lipid metabolism, another key player, influences the formation of myelin and the integrity of neuronal membranes. Dysregulation of lipid metabolism is increasingly associated with neurodevelopmental disorders, including ASDs and attention deficit hyperactivity disorder (ADHD) [10, 11]. Aberrant lipid profiles may impair synaptic function and neural connectivity, contributing to the cognitive and behavioral symptoms observed in these conditions [10]. In addition, the continuum of neurodevelopmental disorders reflects the diverse ways in which metabolic dysregulation manifests across different stages of development. Early disruptions may result in gross structural abnormalities, such as FCD, while later perturbations may primarily affect synaptic function and connectivity [3]. This continuum underscores the importance of early detection and intervention to mitigate long‐term consequences. Furthermore, emerging research highlights the interplay between genetic predisposition and metabolic insults in shaping neurodevelopmental outcomes. For example, mutations in genes encoding metabolic enzymes or mitochondrial proteins can predispose individuals to neurodevelopmental disorders, with environmental factors such as prenatal hypoxia or maternal malnutrition exacerbating their effects [12, 13]. These findings emphasize the need for an integrated approach to studying neurodevelopment that considers both genetic and metabolic contributions.

### 1.4 Toward a Comprehensive Understanding

Advances in high‐throughput technologies, such as single‐cell transcriptomics and metabolomics, are beginning to shed light on the complex interactions between metabolism and neurodevelopment [14]. These tools enable the characterization of cell‐type‐specific metabolic profiles and their dynamic changes during development. Combined with animal models and patient‐derived organoids, these approaches provide unprecedented insights into the mechanisms underlying neurodevelopmental disorders. As our understanding of these relationships deepens, the integration of metabolic insights into neurodevelopmental research holds promise for advancing diagnostics, therapeutics, and preventative strategies. For instance, ketogenic diets, which alter brain metabolism by promoting ketone body utilization, have shown efficacy in managing epilepsy and may hold potential for other neurodevelopmental disorders [5, 15]. Similarly, mTOR inhibitors such as rapamycin are being explored for their ability to correct aberrant signaling in conditions like TSC and FCD [16]. These approaches highlight the therapeutic potential of modulating metabolism to restore neurodevelopmental homeostasis. In this review, we aim to explore the intricate connections between neurodevelopment and metabolism, shedding light on their roles in health and disease.

## 2 Glucose Metabolism

**FIGURE 1:**
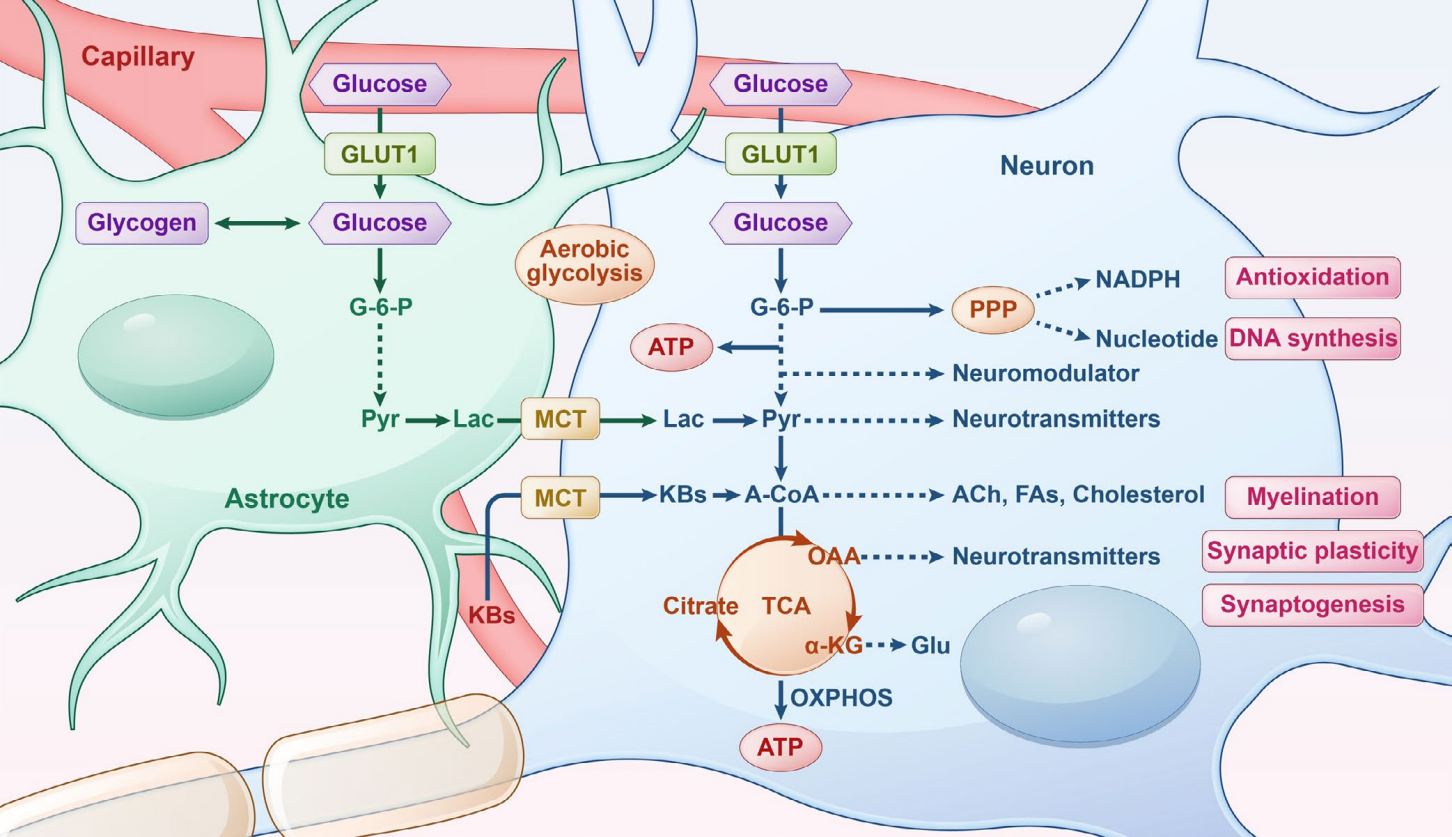
Glucose metabolism in the brain. Glucose enters the brain via glucose transporters and is metabolized in neurons through glycolysis and the pentose phosphate pathway, producing NADPH, nucleotide precursors, and neuromodulators. Pyruvate, oxaloacetate, and α‐ketoglutarate derived from glucose serve as precursors for neurotransmitter synthesis. Astrocytes convert glucose to lactate, which is transported to neurons via monocarboxylate transporters for energy production or biosynthesis. Lactate and ketone bodies generate acetyl‐CoA, fueling ATP synthesis via the TCA cycle and oxidative phosphorylation or supporting the synthesis of acetylcholine, fatty acids, and cholesterol. KBs are preferentially used for FA synthesis. ACh, acetylcholine; A‐CoA, acetyl‐CoA; FAs, fatty acids; G‐6‐P, glucose‐6‐phosphate; Glu, glutamate; GLUT, glucose transporter; KBs, ketone bodies; Lac, lactate; MCT, monocarboxylate transporter; OAA, oxaloacetate; OXPHOS, oxidative phosphorylation; Pyr, pyruvate; TCA, tricarboxylic acid cycle; αKG, α‐ketoglutarate.

Glucose is the principal energy substrate for the brain, which, despite its relatively small size, accounts for a disproportionate share of total body glucose consumption. This metabolic preference reflects the brain's extensive energy demands during neurodevelopment, supporting critical processes such as cell proliferation, differentiation, migration, and synaptic plasticity [17]. Beyond its role as an energy source, glucose provides essential intermediates for biosynthetic pathways, neurotransmitter production, and oxidative stress defense, making it indispensable for brain maturation and function (Figure 1).

### 2.1 Glucose Transport and Cellular Uptake

Glucose utilization in the brain relies on specific glucose transporters (GLUTs), which facilitate its entry across cellular membranes. GLUT1, predominantly expressed in endothelial cells of the BBB, as well as in astrocytes and oligodendrocytes, regulates glucose transport from the bloodstream into the brain parenchyma [18]. In contrast, GLUT3, highly enriched in neurons, ensures their high metabolic demands are met [19]. Once transported into brain cells, glucose undergoes metabolism through two interconnected pathways: OXPHOS via the tricarboxylic acid (TCA) cycle, which facilitates the complete oxidation of glucose to CO_2_, maximizing ATP production, and glycolysis, where glucose is partially oxidized to pyruvate or lactate, providing a rapid energy source and essential biosynthetic precursors. Notably, During neurodevelopment, the brain exhibits a metabolic shift characterized by a greater reliance on aerobic glycolysis (AG), a process where glucose undergoes glycolysis despite sufficient oxygen availability [4]. Unlike in the mature brain, where AG constitutes only 10%–12% of glucose metabolism, its contribution is markedly elevated in the developing brain [20].

### 2.2 Aerobic Glycolysis and Alternative Metabolic Pathways

Aerobic glycolysis plays a critical role in neurodevelopment, not merely as an energy‐producing process but also as a biosynthetic hub (Figure 1). Glucose‐derived intermediates are shunted into the PPP, generating NADPH and nucleotide precursors essential for lipid synthesis, redox homeostasis, and DNA replication [21, 22]. The metabolic byproduct lactate plays diverse roles in brain function. In neurons, lactate serves as a substrate for oxidative metabolism via the TCA cycle, supporting energy production. In oligodendrocytes, lactate promotes myelination, a critical process for axonal insulation and efficient signal propagation [23]. Regions with high AG exhibit elevated expression of genes associated with synaptogenesis and neuronal growth, underscoring its essential role in brain maturation [24]. The synthesis of neurotransmitters (e.g., acetylcholine, glutamate (Glu), gamma‐aminobutyric acid (GABA)) and neuromodulators (e.g., D‐serine, glycine, D‐aspartate) is also closely tied to glucose metabolism [4, 25]. In addition to glucose, the developing brain demonstrates metabolic flexibility by utilizing lactate and ketone bodies (KBs) as alternative energy substrates. Astrocyte‐derived lactate and circulating KBs are transported into neurons via monocarboxylate transporters (MCTs), where they efficiently fuel the TCA cycle [26, 27]. Notably, lactate and KBs utilization peaks during the fetal and early postnatal stages, correlating with high MCT expression and lower levels of GLUT1 and GLUT3 in the neonatal brain [28, 29, 30]. Astrocytes are also important to the brain's metabolic ecosystem. They not only regulate the composition of the interstitial fluid but also serve as reservoirs of glycogen, the brain's primary energy store [31]. During periods of heightened neuronal activity, astrocytes mobilize glycogen to produce lactate, which is subsequently transferred to neurons to sustain energy demands (Figure 1). Additionally, astrocytes recycle neurotransmitters, provide biosynthetic precursors, and mitigate oxidative stress through antioxidant pathways [32]. Together, In the developing brain, glucose metabolism drives energy production, cellular growth, synaptogenesis, and neurotransmitter synthesis. Aerobic glycolysis, lactate, and KBs underscore the brain's metabolic adaptability, with astrocytes playing a key role in supporting neuronal function and development through energy provisioning and regulation.

## 3 Lipid Metabolism

**FIGURE 2:**
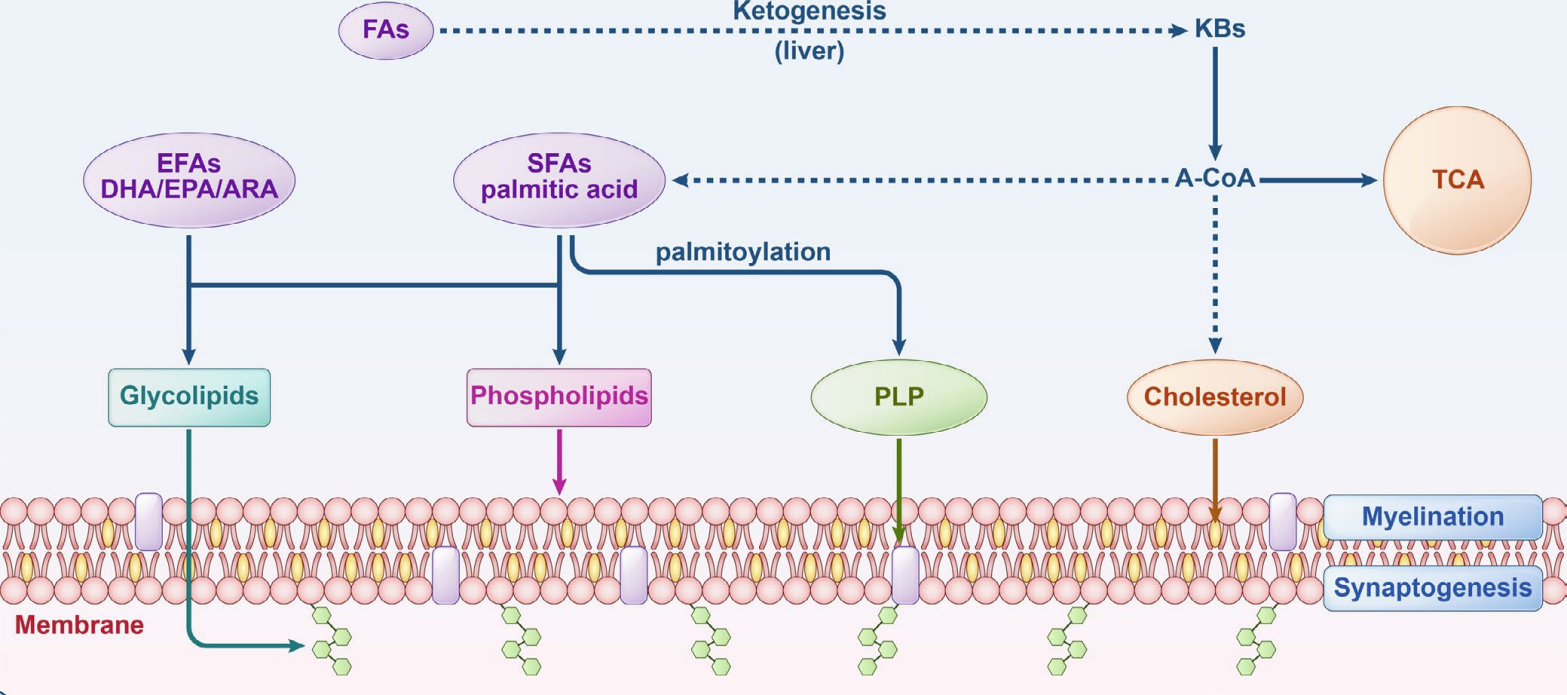
Lipid metabolism in the brain. Saturated fatty acids, starting with palmitic acid, support protein movement within lipid environments. Proteolipid protein is a key component of the central nervous system myelin. Ketone bodies, derived from liver fatty acids, are converted to acetyl‐CoA for lipid synthesis and energy metabolism. Cholesterol maintains membrane integrity and fluidity by integrating into lipid bilayers. Essential fatty acids and SFAs contribute to the synthesis of phospholipids and glycolipids, vital for membrane and myelin structure. A‐CoA, acetyl‐CoA; ARA, arachidonic acid; DHA, docosahexaenoic acid; EFAs, essential fatty acids; EPA, eicosapentaenoic acid; FAs, fatty acids; KBs, ketone bodies; OAA, oxaloacetate; PLP, proteolipid protein; SFAs, saturated fatty acids; TCA, tricarboxylic acid cycle; αKG, α‐ketoglutarate.

Lipids form the essential foundation of brain tissue, accounting for approximately 78% of the dry weight of the axon myelin sheath and 35%–40% of the neuron‐rich gray matter [33]. These biomolecules, encompassing saturated fatty acids (SFAs), unsaturated fatty acids (UFAs), cholesterol, sterols, and complex lipids, are instrumental in numerous critical neurodevelopmental processes, particularly myelination, the intricate formation of myelin sheaths that insulate nerve fibers and enhance the rapid transmission of neural signals (Figure 2).

### 3.1 Fatty Acids

Fatty acids can be categorized into SFAs and UFAs. SFAs are primarily synthesized within the human body, with palmitic acid (hexadecanoic acid) serving as the initial substrate in fatty acid biosynthesis (Figure 2). Subsequent fatty acids are produced through modifications of palmitic acid occurring in the endoplasmic reticulum and mitochondria. Beyond its structural role in nervous tissue, palmitic acid is functionally significant, facilitating the movement of proteins within lipid environments through a process known as palmitoylation [34, 35]. The myelin proteolipid protein (PLP), a principal component of myelin, contains approximately equal proportions of palmitic acid and stearic acid [35]. During critical early developmental stages, newly formed synapses may modulate the palmitoylation of specific axon growth‐associated proteins, such as GAP‐43, potentially reducing its palmitoylation [34, 36]. Thus, palmitic acid is implicated in various biological processes, including palmitoylation, gliogenesis, synaptogenesis, and myelination.

UFAs can be further distinguished into monounsaturated fatty acids (MUFAs) and polyunsaturated fatty acids (PUFAs). PUFAs are classified based on the position of their first double bond into omega‐3 (ω‐3), omega‐6 (ω‐6), and omega‐9 (ω‐9) [37]. Among these, essential fatty acids (EFAs), which the human body cannot synthesize and must be derived from the diet, include alpha‐linolenic acid (ALA) and linoleic acid (LA). ALA, a precursor for ω‐3 fatty acids, can be metabolized into docosahexaenoic acid (DHA) and eicosapentaenoic acid (EPA), while LA serves as a precursor for ω‐6 arachidonic acid (ARA) [37]. DHA and ARA constitute approximately 20% of the fatty acids within brain tissue, forming vital components of cell membrane lipids [10] (Figure 2). These fatty acids intercalate with phospholipids, promoting neural development, myelination, and neural repair [38, 39]. Elevated levels of DHA in synaptosomes are known to foster neurite outgrowth and synaptogenesis [40], while DHA also plays a crucial role in the membranes of glial cells, regulating myelin formation in oligodendrocytes [41]. Notably, supplementation of DHA and ARA in pregnant and breastfeeding mothers has been linked to improved visual function in infants, and the delicate balance between these fatty acids may significantly influence cognitive development in children [42, 43]. Furthermore, supplementation of ω‐3 PUFAs has shown promise in enhancing cognitive function in healthy children and adolescents, with potential benefits for those diagnosed with ASD or ADHD [11, 44].

### 3.2 Complex Lipids, Cholesterol and Ketone Bodies

Complex lipids, including phospholipids and glycolipids, are equally pivotal for myelination. Sphingomyelin, a prominent phosphorylated sphingolipid, constitutes a major component of the myelin sheath, while gangliosides facilitate neurotransmitter binding to synaptic membranes, thereby enhancing synaptic function and neurotransmission. The maturation of the brain is closely associated with an increase in ganglioside levels, particularly during the prenatal and early postnatal periods [12, 45].

Cholesterol, a vital lipid component, is intricately embedded within the lipid bilayer of cell membranes, particularly in myelin. Cholesterol is mainly absorbed from food, and it can also be synthesized from acetyl coenzyme A (acetyl‐CoA). It plays a critical role in maintaining membrane structural integrity and fluidity [46, 47], which are essential for neuronal differentiation and the formation of dendrites and axons. In myelin, cholesterol comprises approximately 40% of the lipid content, significantly contributing to the insulating properties of the myelin sheath and enhancing the velocity of nerve impulse conduction through saltatory conduction [48, 49]. Furthermore, cholesterol acts as a precursor for steroid hormones, influencing both embryonic and postnatal brain development through epigenetic mechanisms such as DNA methylation and histone modifications [50].

Ketone Bodies, comprising acetoacetate, acetone, and β‐hydroxybutyrate, are particularly crucial during fetal development [51]. These metabolic derivatives of fatty acids are transported into the brain via monocarboxylate transporter 1 (MCT1) and are converted into acetyl‐CoA within mitochondria, playing an indispensable role in lipid synthesis and energy metabolism. While glucose metabolism may decline with age, the metabolism of KBs remains consistent, although their distribution varies across different brain regions [5].

Consequently, lipid metabolism serves as a cornerstone for the development and maintenance of brain structure and function. By providing essential substrates for myelin synthesis and modulating oligodendrocyte function, lipids play a pivotal role in myelination, an intricate process vital for the efficient transmission of neural signals and the overarching maturation of the brain.

## 4 Amino Acid Metabolism

**FIGURE 3:**
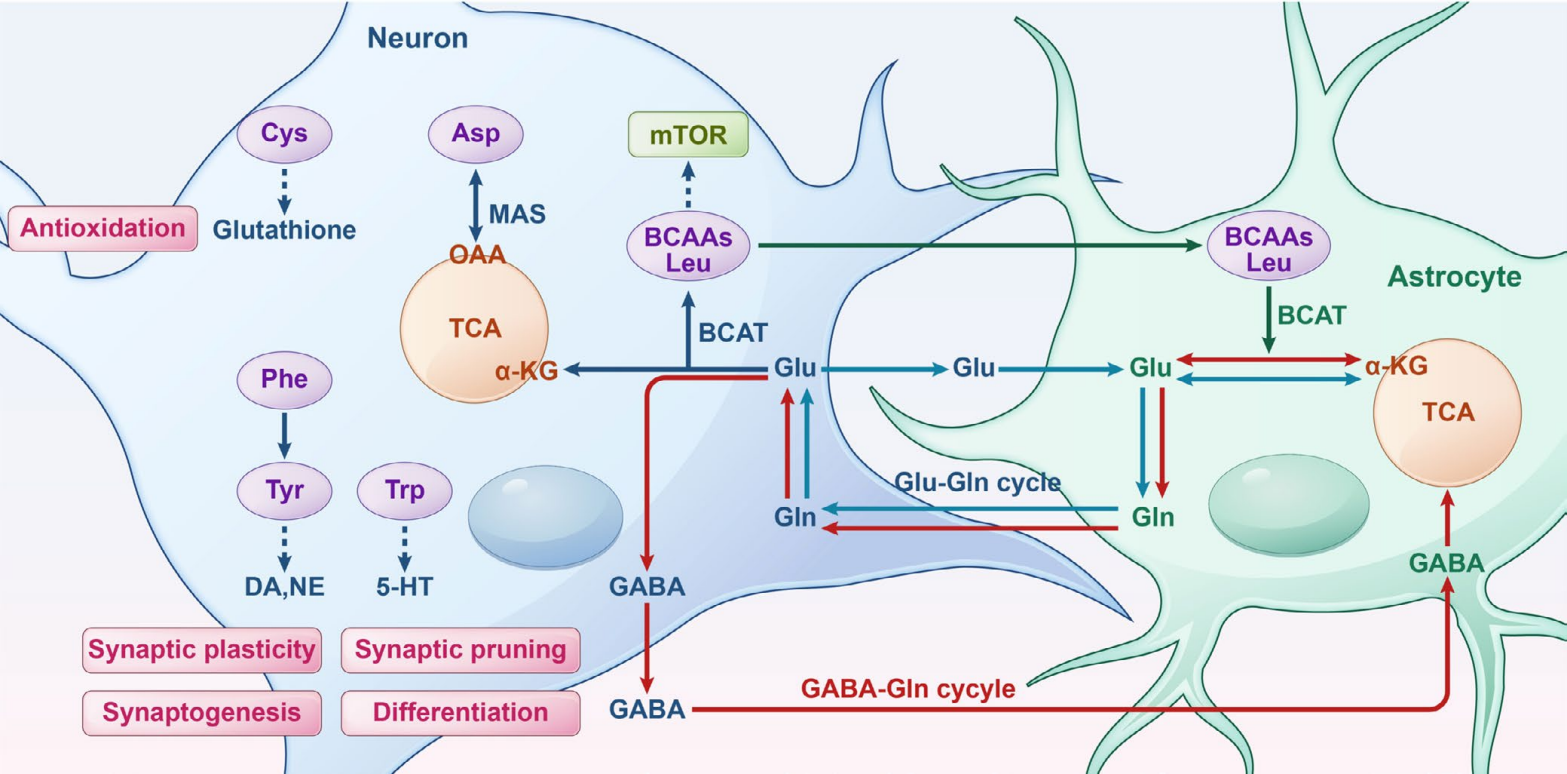
Amino acid metabolism in neurotransmitter and antioxidant synthesis. The glutamate/GABA‐glutamine cycle regulates excitatory and inhibitory signaling, essential for neurodevelopment. Glu is synthesized de novo in astrocytes, while branched‐chain amino acids act as nitrogen donors for neurotransmitter synthesis, with leucine activating the mTOR pathway. Phenylalanine, tyrosine, and tryptophan serve as precursors for neurotransmitters such as dopamine, norepinephrine, epinephrine, and serotonin. Cysteine is a precursor for the antioxidant glutathione, and aspartate supports mitochondrial electron transport through the malate–aspartate shuttle. 5‐HT, serotonin; BCAAs, branched‐chain amino acids; BCAT, branched‐chain aminotransferases; DA, dopamine; GABA, gamma‐aminobutyric acid; Glu, glutamate; MAS, malate–aspartate shuttle; NE, norepinephrine; OAA, oxaloacetate; TCA, tricarboxylic acid cycle; αKG, α‐ketoglutarate.

Amino acids are essential not only as building blocks for protein synthesis but also as precursors for neurotransmitters, playing a critical role in neuronal growth, synaptic plasticity, and overall brain function. Disruptions in amino acid metabolism can disturb neurotransmitter balance, impairing cognitive processes, behavior, and neural development (Figure 3).

### 4.1 The Glutamate/GABA‐Glutamine Cycle

Glutamate, the brain's primary excitatory neurotransmitter, is essential for driving learning, memory, and synaptic plasticity. In contrast, GABA, the principal inhibitory neurotransmitter, ensures stability within neural circuits by dampening neuronal excitability. Notably, during early brain development, GABA initially functions in an excitatory capacity before transitioning to its inhibitory role as the brain matures [52]. Neurons rely on astrocytes to supply precursors for glutamate and GABA, as they cannot synthesize these neurotransmitters directly from glucose [53]. In astrocytes, glutamine is derived from α‐ketoglutarate and transported into neurons, where it is converted into either glutamate or GABA. Following synaptic release, these neurotransmitters are reabsorbed by astrocytes, with up to 85% recycled back into glutamine [53, 54]. This tightly coordinated cycle (Figure 3) not only links neurotransmitter activity to glucose metabolism but also demonstrates the metabolic interplay between neurons and glia, as glutamate uptake by astrocytes stimulates glycolysis [55]. Moreover, glutamatergic signaling is crucial for astrocyte differentiation [56], reflecting the close interplay between neurons and glia. The balance between glutamate and GABA is fundamental to neural circuit activity and plasticity. Glutamate drives excitatory synaptic plasticity, such as long‐term potentiation (LTP), which is critical for learning and memory [57], while GABA regulates inhibitory synaptic plasticity, like long‐term depression (LTD), to refine neural circuits [58]. Both neurotransmitters also guide synaptic pruning during brain maturation [59, 60]. Disruption of this delicate balance can impair neural connectivity and contribute to neurodevelopmental disorders.

### 4.2 Tryptophan Tyrosine and Branched‐Chain Amino Acids (BCAAs)

In addition to these neurotransmitters, tryptophan and tyrosine are essential amino acids that serve as precursors for critical neurotransmitters involved in neurodevelopment. Tryptophan is converted into serotonin (5‐HT), a neurotransmitter that regulates mood, sleep, memory, and behavior. During early brain development, serotonin is indispensable for processes such as neuronal differentiation, synapse formation, and the establishment of functional neural circuits. This neurotransmitter is not only crucial for neural development but also influences the timing of developmental milestones. Abnormal serotonin signaling during these critical periods has been linked to neurodevelopmental disorders, including ASD [56]. Similarly, tyrosine, derived from phenylalanine or dietary proteins, is a precursor for catecholamines, which include dopamine (DA), norepinephrine (NE), and epinephrine. Dopamine plays a central role in motor control, reward processing, and cognitive function, while catecholamines broadly influence neural circuit maturation, synaptic plasticity, and emotional regulation. This catecholaminergic system is vital for adapting to environmental challenges and experiences. Dysregulation in these pathways is associated with a range of psychiatric and neurological disorders, such as ADHD, Parkinson's disease, and schizophrenia [61, 62, 63, 64].

In addition to these neurotransmitter precursors, BCAAs, such as leucine, isoleucine, and valine, act as vital nitrogen donors for the synthesis of neurotransmitters like glutamate and glutamine [65]. Leucine, in particular, activates the mTOR pathway, a master regulator of cell growth, proliferation, and metabolism, regulating neuronal differentiation and synapse formation, which are crucial for developing complex neural networks during brain development [66]. Furthermore, neurodevelopment is highly sensitive to oxidative stress, which can trigger apoptosis and compromise neuronal survival. Amino acids such as cysteine play a protective role by serving as precursors for glutathione, a potent antioxidant that shields neurons from oxidative damage and helps maintain cellular integrity during critical developmental periods. The malate–aspartate shuttle (MAS) transfers high‐energy electrons from cytoplasmic NADH to mitochondria for ATP production. Aspartate is essential in this process, facilitating transamination reactions that interconvert oxaloacetate and malate and acting as a carrier between the cytoplasm and mitochondria to sustain the shuttle cycle [4]. This transfer ensures a continuous electron flow into the mitochondrial electron transport chain, vital for efficient energy production. Maintaining the balance between oxidative and reductive states is critical for proper neuronal function and survival [4].

Together, these amino acids and their derivatives not only contribute to the synthesis of key neurotransmitters but also play pivotal roles in ensuring proper neurodevelopment and protecting against neurodevelopmental disorders, illustrating the intricate biochemical interplay necessary for healthy brain maturation [4].

## 5 Mitochondria

### 5.1 Essential Organelles in Energy Production

Mitochondria are indispensable organelles in neurodevelopment, serving as central hubs for energy production, cellular metabolism, and the regulation of cell death. Their primary function, generating ATP through OXPHOS, is particularly critical in the CNS [67, 68].

### 5.2 Mitochondria's Role in Brain Development

Beyond their bioenergetic role, mitochondria are intimately involved in the processes that drive brain development. During neurogenesis, mitochondrial function supports neural stem cell proliferation, differentiation, and maturation, as well as the formation of dendrites and axonal projections. By dynamically adjusting their shape and distribution through fusion, fission, and biogenesis, mitochondria ensure energy delivery to areas of high demand, such as growing dendritic spines and synaptic terminals [6]. This ability to modulate energy production and distribution is essential for synaptic development, plasticity, and the refinement of neural circuits [6, 69, 70, 71, 72]. Mitochondria are also key regulators of redox signaling. While ROS, natural byproducts of OXPHOS, are often associated with oxidative stress, at physiological levels they function as signaling molecules that influence processes like neuronal differentiation, axonal guidance, and synaptic remodeling [6]. However, mitochondrial dysfunction can lead to excessive ROS accumulation, causing oxidative damage to lipids, proteins, and DNA, ultimately compromising neuronal survival and brain development [6]. In addition, mitochondria are involved in apoptosis, a form of programmed cell death essential for refining neurodevelopment. Apoptosis ensures the elimination of excess, damaged, or improperly integrated neurons, thereby sculpting functional neural networks and maintaining cellular balance [71]. Dysregulation of mitochondrial‐mediated apoptosis, however, can disrupt this delicate equilibrium, resulting in either excessive neuronal loss or inappropriate survival, which are hallmarks of various neurodevelopmental disorders [71, 73]. Thus, the maintenance of mitochondrial health, including their metabolic capacity, dynamics, and redox balance, is fundamental for proper neurodevelopment. Dysfunctional mitochondria, whether due to genetic mutations, environmental insults, or metabolic imbalances, are increasingly recognized as contributors to neurodevelopmental disorders, such as ASD, epilepsy, and intellectual disabilities.

## 6 Metabolic Pathways Shaping Neurodevelopment

Neurodevelopment is intricately governed by key metabolic pathways, such as insulin, mTOR, and adenosine monophosphate‐activated protein kinase (AMPK) signaling, which coordinate processes like synaptic plasticity, neuronal growth, and energy balance. These pathways integrate molecular cues to regulate autophagy, stress responses, and cellular metabolism. Additionally, epigenetic modifications shaped by metabolic intermediates play a vital role in fine‐tuning gene expression, emphasizing their significance in brain development and potential therapeutic strategies.

### 6.1 Key Metabolic Pathways in Neurodevelopment

Insulin signaling primarily regulates glucose, lipid, and protein metabolism by activating the mitogen‐activated protein kinase (MAPK) and the phosphatidyl‐inositol 3‐kinase (PI3K)/protein kinase B (Akt) pathways via the insulin receptor (IR), which is crucial for neuronal growth, differentiation, and survival during neurodevelopment [74]. The MAPK pathway is involved in synaptic growth, neuron maintenance, and repair, while the PI3K/Akt pathway plays a key role in maintaining synaptic plasticity, stress response, neuronal metabolism, and autophagy [75, 76]. Insulin‐like growth factor 1 (IGF1) signaling is essential for early brain development. IGF1 receptor (IGF1R) is similar to IR, acting as tyrosine kinase receptors [77]. IGF1 exerts diverse effects across all neural cell types, including neurons, oligodendrocytes, and astrocytes, by promoting cell proliferation and maturation, as well as enhancing myelination [78]. At both tissue and cellular levels, IGF1 signaling has widespread impacts on metabolism, affecting glucose and lipid homeostasis and protein turnover [79]. Insulin‐like growth factor 2 (IGF2) is important for protein transport, synthesis, and degradation through its receptor, contributing actively to neuronal plasticity and the formation and enhancement of memory [80]. IGF2 is targeted by the IκB kinase (IKK) /neuron‐specific nuclear factor κB (NF‐κB) pathway via the IGF2 receptor (IGF2R), which is crucial for synapse formation and maturation [80]. IGF1 and IGF2 share significant homology with each other and with proinsulin. Due to their structural similarity, insulin, IGF1, and IGF2 can cross‐bind to each other's receptors with varying, generally lower, affinities. However, insulin does not appear to bind IGF2R [77, 81]. The mTOR pathway is a critical regulator of cell growth and metabolism, integrating signals from nutrients, growth factors, and cellular energy status to modulate neuronal development and plasticity. For instance, mammalian target of rapamycin complex 1 (mTORC1) activates key lipid regulators, such as sterol regulatory element binding proteins (including SREBP1a, SREBP1c, and SREBP2), leading to increased transcription of genes involved in fatty acid and cholesterol biosynthesis [82, 83]. Aberrant mTOR signaling is implicated in various neurodevelopmental disorders, including TSC, FCD, and Rett syndrome [16, 84]. Ongoing research into mTOR inhibitors and modulators aims to explore their therapeutic potential for managing neurodevelopmental disorders and promoting brain health [9, 85, 86]. AMPK serves as a cellular energy sensor, maintaining energy homeostasis during neurodevelopment, particularly under metabolic stress. When energy levels are low (high AMP/ATP ratio), AMPK activation inhibits anabolic processes and stimulates catabolic pathways to restore ATP levels [87]. It also regulates mitochondrial biogenesis, fission, and autophagy, making it a central modulator of mitochondrial homeostasis [88]. Notably, there is a co‐regulation between the AMPK and mTOR pathways [88]. Energy‐sensing neurons in the hypothalamus help regulate overall energy balance through AMPK signaling. AMPK activation in the hypothalamus is influenced by energy‐related signals linked to food intake (fasting and feeding), energy expenditure (ATP levels), and body weight (hormonal and adipose factors) [88]. Together, these key signaling pathways orchestrate neurodevelopment by regulating neuronal growth, metabolism, plasticity, and energy homeostasis, while dysregulation of these pathways is linked to neurodevelopmental disorders and metabolic stress.

### 6.2 Epigenetic Modifications

Epigenetic modifications, such as DNA methylation and histone modification, are influenced by metabolic intermediates and nutrients. One‐carbon metabolism, which includes the folate cycle, methionine cycle, and transsulfuration pathway, is essential for the biosynthesis of nucleic acids, proteins, and lipids, as well as for epigenetic modifications that regulate DNA methylation and gene expression [89]. Disruptions in these pathways can lead to abnormal epigenetic changes, adversely affecting neurodevelopment and increasing the risk of developmental disorders. For instance, folate deficiency during pregnancy is associated with neural tube defects, underscoring the importance of adequate nutrient intake for proper brain development. Research into the role of methyl donors in epigenetic regulation further elucidates how metabolic factors can influence gene expression and neurodevelopment [89, 90]. Understanding these interactions can inform dietary recommendations and interventions aimed at supporting optimal brain development.

## 7 Metabolic Dysregulation and Its Impact on Neurodevelopmental Disorders

The interplay between metabolism and neurodevelopment is complex and bidirectional. Metabolic processes supply the essential substrates and energy for brain development, while the brain's metabolic state significantly influences developmental outcomes. Disruptions or deficiencies in these processes can derail normal brain development, giving rise to various neurodevelopmental disorders. For instance, abnormalities in glucose metabolism are linked to cognitive impairments and developmental delays, while lipid metabolism defects can impair myelination and neuronal function [5, 49]. Similarly, imbalances in amino acid metabolism, as seen in conditions like phenylketonuria and maple syrup urine disease, significantly hinder brain development and function [65, 91]. Recent advances in genomics, proteomics, and metabolomics have deepened our understanding of these mechanisms, enabling the identification of metabolic biomarkers and therapeutic targets for neurodevelopmental disorders. Techniques such as next‐generation sequencing (NGS) and mass spectrometry have uncovered molecular disruptions underlying these conditions. Furthermore, studies of epigenetic modifications, including DNA methylation mediated by one‐carbon metabolism, illustrate how nutrient availability shapes gene expression and developmental trajectories [89, 90]. Recognizing the role of metabolic dysregulation in neurodevelopmental disorders is pivotal for developing effective treatments. Targeted interventions, such as dietary modifications and pharmacological therapies, can help restore metabolic balance and improve outcomes. Additionally, understanding metabolic influences on neurodevelopment provides a foundation for preventive strategies and early interventions to mitigate the risk of these disorders in vulnerable populations (Table 1).

**TABLE 1:** Neurodevelopmental disorders.

Metabolic dysfunction	Diseases	Biology impairments
Glucose	GLUT1 deficiency syndrome, PAST‐A	Glucose transport
	MCT1 deficiency	Lactate and KBs transport
	MCT12 deficiency	Creatine transport
	TPI deficiency, RPI deficiency, and TKT deficiency	Glycolysis and PPP
Lipids	Sphingolipidoses (Krabbe disease, Gaucher disease, Tay–Sachs disease, Niemann‐Pick disease)	Sphingolipid metabolism
	SLOS, MKD(MVA/HIDS)	Sterol metabolism
Animo acids	PKU	Phenylalanine metabolism
	MSUD	BCAAs metabolism
	CCDs (CTD, AGAT deficiency, GAMT deficiency)	Creatine biosynthesis and transport
Mitochondria	Leigh syndrome, MELAS, LHON	OXPHOS
Multifaceted	ASD	Mitochondria, Amino acids and lipids metabolism, Gut microbiota
	ADHD	Amino acids and lipids metabolism, Gut microbiota

Abbreviations: ADHD, attention deficit hyperactivity disorder; AGAT, arginine, glycine amidinotransferase; ASD, autism spectrum disorder; BCAAs, branched‐chain amino acids; CCDs, cerebral creatine deficiencies; CTD, creatine transporter deficiency; GAMT, guanidinoacetate methyltransferase; GLUT, glucose transporter; HIDS, hyper‐immunoglobulinemia D and periodic fever syndrome; LHON, Leber hereditary optic neuropathy; MCT, monocarboxylate transporter; MELAS, mitochondrial encephalomyopathy, lactic acidosis, and stroke‐like episodes; MKD, mevalonate kinase deficiency; MSUD, maple syrup urine disease; MVA, mevalonic aciduria; OXPHOS, oxidative phosphorylation; PKU, phenylketonuria; RPI, ribose 5‐phosphate isomerase; SLOS, Smith–Lemli–Opitz syndrome; TKT, transketolase; TPI, triosephosphate isomerase.

### 7.1 Glucose Metabolic Dysregulation and Associated Neurodevelopmental Disorders

Glucose serves as the primary energy substrate for the brain, and its dysregulation can lead to severe developmental consequences. Congenital hyperinsulinism exemplifies how excessive insulin production causes recurrent hypoglycemia, leading to developmental delays and cognitive impairments [92]. Similarly, glucose transporter deficiencies, such as GLUT1 deficiency syndrome, impair glucose uptake in the brain, resulting in seizures, developmental delays, and movement disorders [93]. These conditions highlight the necessity of tightly regulated glucose supply for normal brain function. Monogenic disorders affecting glucose transport and metabolism, such as those involving proton‐associated sugar transporter A (*SLC45A1*), MCT1 (*SLC16A1*), and MCT12 (*SLC16A12*), can disrupt the transportation of glucose and its metabolites. These disruptions manifest as developmental delays, behavioral disturbances, seizures, and movement disorders [5]. Additionally, defects in glycolysis and the PPP, such as triosephosphate isomerase (TPI) deficiency, ribose 5‐phosphate isomerase (RPI) deficiency, and transketolase (TKT) deficiency, predominantly affect the CNS and are associated with poor therapeutic outcomes due to limited treatment options [5]. Glycogen metabolism disorders further illustrate the impact of glucose dysregulation on neurodevelopment. Dysregulation in glycogen synthesis or catabolism leads to the accumulation of abnormal glycogen structures, as seen in Lafora disease, which is characterized by severe seizures and early mortality [94]. Furthermore, recent studies have also linked chronic abnormalities in glucose metabolism to structural and functional changes in the brain. For instance, type 1 diabetes (T1D) has been associated with hippocampal dysfunction, impairing learning and memory processes [4, 95]. Chronic hypoglycemia can trigger neuronal apoptosis and cognitive deficits, while hyperglycemia may adversely affect neurogenesis and plasticity [30, 95, 96, 97]. These findings highlight the delicate balance required in glucose metabolism to support normal neurodevelopment and prevent neurological complications. When glucose availability is insufficient, KBs serve as an alternative energy source, particularly during childhood. The ketogenic diet (KD), which promotes the production of 4‐carbon KBs, has demonstrated efficacy in managing GLUT1 deficiency syndrome and other forms of intractable epilepsy, with approximately 60% of patients achieving seizure freedom [15, 98, 99]. This underscores the potential of energy metabolism‐based therapies in treating neurodevelopmental disorders.

### 7.2 Neurodevelopmental Disorders Associated With Lipid Metabolism Disruption

Abnormalities in lipid metabolism are implicated in several neurodevelopmental and neurological disorders. Sphingolipidoses, including Niemann‐Pick disease, Gaucher disease, Tay–Sachs disease, and Krabbe disease, arise from defective sphingolipid metabolism. These disorders lead to the accumulation of lipids in various tissues, particularly the brain, resulting in neurodegeneration, disrupted myelin formation, and severe neurological impairments [100, 101]. Similarly, disorders such as Smith–Lemli–Opitz syndrome (SLOS) and mevalonate kinase deficiency (including mevalonic aciduria and the milder hyper‐immunoglobulinemia D and periodic fever syndrome, or HIDS) are caused by mutations in enzymes (*DHCR7* and *MVK*) involved in sterol metabolism. These mutations contribute to a diverse range of characteristic CNS malformations [47]. In addition to these specific disorders, the role of essential fatty acids, particularly omega‐3 and omega‐6 fatty acids, has gained significant attention in relation to brain development. Omega‐3 fatty acids, especially DHA, are crucial for neuronal growth and synaptic plasticity. Deficiencies in these fatty acids can impair cognitive function and elevate the risk of neurodevelopmental disorders. Research indicates that omega‐3 supplementation can enhance cognitive outcomes in children with developmental delays and in individuals with neurodegenerative diseases [10].

### 7.3 Amino Acid Metabolism and Its Role in Neurodevelopmental Disorders

A variety of metabolic disorders related to amino acid metabolism significantly impact neurodevelopment. One of the most well‐known disorders is phenylketonuria (PKU), which results from a deficiency in the enzyme phenylalanine hydroxylase. This deficiency leads to the accumulation of phenylalanine, which can cause cognitive impairments and developmental delays if not managed through strict dietary restrictions [91, 102]. Another important condition is maple syrup urine disease (MSUD), which affects the metabolism of branched‐chain amino acids. If left untreated, MSUD can lead to severe neurological symptoms and long‐term cognitive deficits [103]. Cerebral creatine deficiencies (CCDs) also highlight the critical role of amino acids in neurodevelopment. Creatine, derived from glycine and arginine, is essential for storing and transmitting phosphate‐bound energy in the brain and muscles. Disorders such as creatine transporter deficiency (CTD), arginine: glycine amidinotransferase (AGAT) deficiency, and guanidinoacetate methyltransferase (GAMT) deficiency disrupt the transportation and metabolism of creatine, resulting in developmental delays, regression, intellectual disability, and difficulties with expressive and cognitive speech. Notably, creatine supplementation has been shown to alleviate some of these symptoms [104, 105].

### 7.4 Mitochondrial Dysfunction in Neurodevelopment

Mitochondrial dysfunctions are increasingly recognized as significant contributors to both neurodevelopmental and neurodegenerative disorders. Defects in the OXPHOS system are implicated in several conditions, including Leigh syndrome, Leber hereditary optic neuropathy (LHON), and mitochondrial encephalomyopathy, lactic acidosis, and stroke‐like episodes (MELAS). These disorders often result in developmental delays, cognitive impairments, and neurological degeneration, underscoring the critical role of mitochondrial health in neuronal development and function [7]. Moreover, emerging evidence suggests a relationship between mitochondrial dysfunction and ASDs [106]. Recent research has focused on exploring therapeutic strategies to mitigate the effects of mitochondrial dysfunction. These strategies include the use of mitochondrial‐targeted antioxidants and metabolic modulators, which aim to enhance mitochondrial function and provide neuroprotective effects. Such approaches hold promise for developing new therapeutic avenues for neurodevelopmental and neurodegenerative disorders [7, 106, 107].

### 7.5 The Gut‐Brain Axis as a Mediator of Neurodevelopment and Metabolism

In recent years, there has been a growing recognition of the complex interplay between the gut microbiota, metabolism, and the central nervous system, underscoring the significance of the gut‐brain axis (GBA) as a crucial communication network. This axis not only influences neurological health and cognitive function but also plays a vital role in regulating metabolic processes. Through various mechanisms, the GBA facilitates a dynamic interaction between the gut microbiota and the brain, impacting both brain development and metabolic regulation.

**FIGURE 4:**
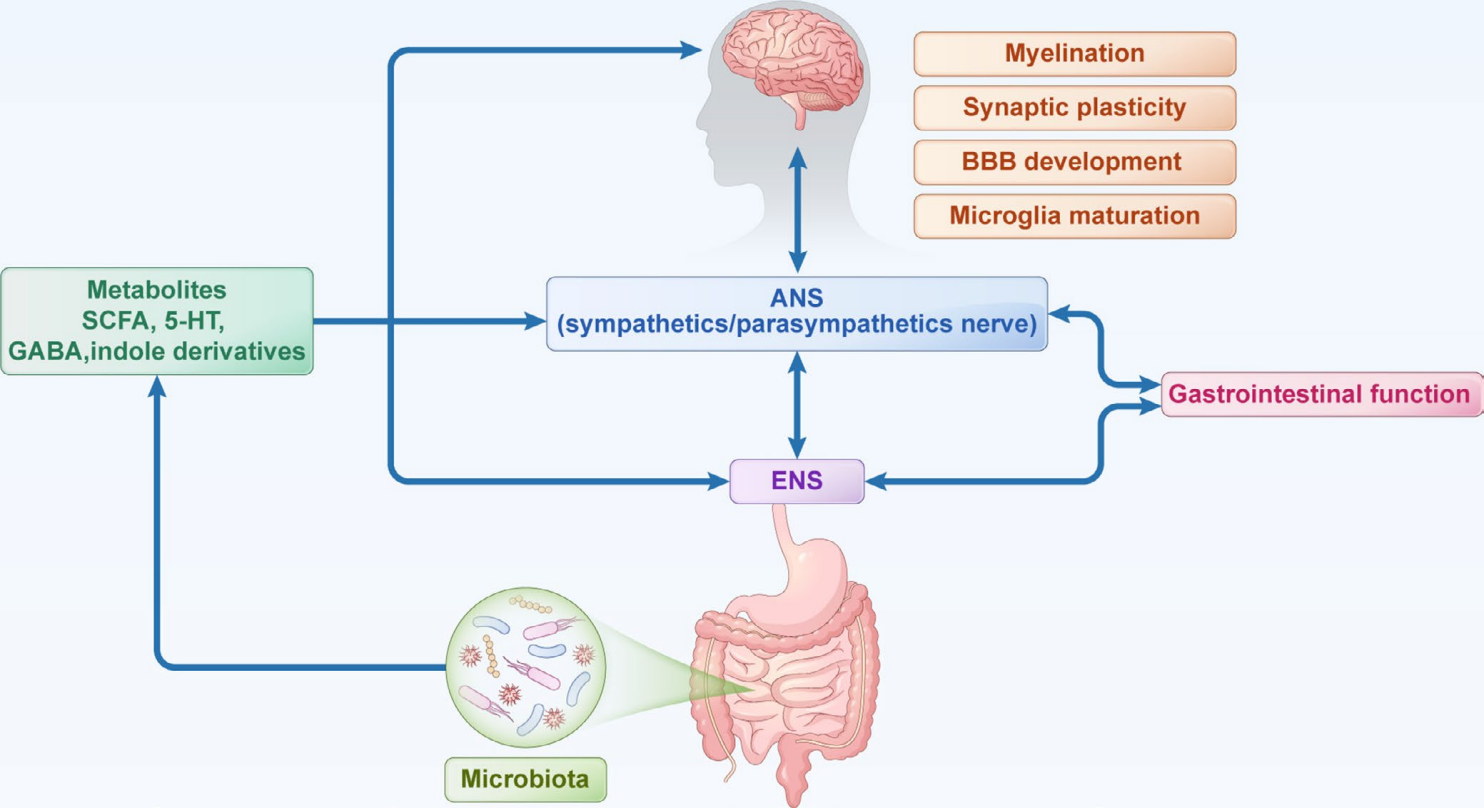
Gut microbiota metabolites regulate neurodevelopment. Gut microbiota and their metabolites, such as SCFAs, secondary bile acids, 5‐HT, and GABA, influence neurodevelopment directly via the circulatory system and indirectly through modulation of the ENS and ANS. Conversely, the brain also regulates gastrointestinal function through the ANS and ENS. 5‐HT, serotonin; ANS, autonomic nervous system; BBB, blood–brain barrier; ENS, enteric nervous system; GABA, gamma‐aminobutyric acid.; SCFA, short‐chain fatty acid.

GBA is a bidirectional communication network linking the gut microbiota and the brain through neural, endocrine, and immune pathways [108]. It primarily involves the central nervous system (CNS), autonomic nervous system (ANS), enteric nervous system (ENS), and the hypothalamic–pituitary–adrenal (HPA) axis. Given the crucial role of gut microbiota in this interaction, the concept of the microbiota‐gut‐brain axis (MGB) has been introduced [109]. Beyond regulating host metabolism, the gut microbiota significantly influences brain development and function [109]. Here, we focus on the neural pathways involved in neurodevelopment (Figure 4). Gut microbiota and their metabolites, such as short‐chain fatty acids (SCFAs) (all the abbreviations are listed in Table 2), secondary bile acids, indole derivatives, serotonin, and GABA, modulate neuropeptide production via endothelial and enteroendocrine cells or directly influence neurotransmitter signaling [110, 111]. SCFAs (acetate, propionate, butyrate), produced by bacterial fermentation of dietary fibers, serve as energy sources for neurons and regulate immune responses [112]. Preclinical evidence suggests that SCFAs modulate BBB development and maintenance via epigenetic modifications [108, 109, 110, 111, 112, 113]. Gut microbes also regulate the metabolism of tryptophan, phenylalanine, and glutamate, which are precursors for essential neurotransmitters like serotonin, dopamine, and GABA, all of which are critical for neurodevelopment [112]. Additionally, microbiota influence brain‐derived neurotrophic factor (BDNF), synaptophysin, and postsynaptic density proteins, key regulators of brain development and plasticity [114, 115]. Microbial‐derived metabolites such as indoles, phenolic acids, and lipopolysaccharides (LPS) can cross the BBB, impacting neuronal function and neuroinflammation [109, 113]. Studies indicate that gut microbiota regulate neurogenesis, cortical myelination, dendritic development, and microglial maturation, highlighting their essential role in brain structure and function [116, 117, 118].

**TABLE 2:** Abbreviations.

Abbreviations	Full terms
GLUT	Glucose transporter
MCT	Monocarboxylate transporter
TPI	Triosephosphate isomerase
RPI	Ribose 5‐phosphate isomerase
TKT	Transketolase
SLOS	Smith–Lemli–Opitz syndrome
MKD	Mevalonate kinase deficiency
MVA	Mevalonic aciduria
HIDS	Hyper‐immunoglobulinemia D and periodic fever syndrome
PKU	Phenylketonuria
MSUD	Maple syrup urine disease
CCDs	Cerebral creatine deficiencies
CTD	Creatine transporter deficiency
AGAT	Arginine: glycine amidinotransferase
GAMT	Guanidinoacetate methyltransferase
BCAAs	Branched‐chain amino acids
MELAS	Mitochondrial encephalomyopathy, lactic acidosis, and stroke‐like episodes
LHON	Leber hereditary optic neuropathy
OXPHOS	Oxidative phosphorylation
ASD	Autism spectrum disorder
ADHD	Attention deficit hyperactivity disorder
PLP	Proteolipid protein
DHA	Docosahexaenoic acid
ARA	Arachidonic acid
EPA	Eicosapentaenoic acid
MAS	Malate–aspartate shuttle
DA	Dopamine
NE	Norepinephrine
5‐HT	Serotonin
GABA	Gamma‐aminobutyric acid
ANS	Autonomic nervous system
ENS	Enteric nervous system
BBB	Blood–brain barrier
SCFA	Short‐chain fatty acid

Disruptions in gut microbiota composition (dysbiosis), an imbalance in gut microbiota composition, have been linked to several neurodevelopmental disorders, including ASDs and ADHD. Research indicates that individuals with ASDs often exhibit altered gut microbiota, with changes in populations of Bifidobacteria, Lactobacilli, Clostridia, Bacteroides, and Firmicutes [119]. This imbalance may contribute to dysregulation of SCFA metabolism, gut barrier dysfunction (“leaky gut”), and chronic inflammation, all of which can impact brain function [120]. Similarly, ADHD is associated with reduced microbial diversity, with certain bacterial species, such as Prevotella, implicated in dopamine regulation, suggesting a role for gut microbiota in neurotransmitter modulation [121]. Targeting gut microbiota through interventions like probiotics, dietary modifications, and fecal microbiota transplantation (FMT) holds promise for therapeutic applications. Studies suggest that supplementing with probiotics and prebiotics can improve symptoms of ASDs and ADHD by enhancing SCFA production and regulating neurotransmitter levels [119]. Additionally, FMT has shown potential in alleviating autism‐related behavioral symptoms [120].

### 7.6 ASDs and Metabolic Dysfunctions

Neurodevelopmental disorders are marked by diverse and complex challenges in brain development and function. These conditions often arise from intricate interactions between genetic predispositions, environmental factors, and metabolic dysfunctions, profoundly influencing neurological, cognitive, and behavioral outcomes. Notably, the interaction between these disorders and metabolic dysfunctions typically involves multiple metabolic pathways and mechanisms. ASDs serve as a prominent example, which we explore in greater detail here.

#### 7.6.1 ASDs

ASDs present a broad and heterogeneous range of manifestations, characterized primarily by persistent deficits in social communication and interaction, alongside restricted and repetitive patterns of behavior and interests [107, 122]. Common comorbidities include various neurological, psychiatric, and physical conditions. Neurological issues may encompass epilepsy, sleep disorders, sensory abnormalities, and motor delays or impairments [122]. Psychiatric conditions often involve depression, anxiety, irritability, and ADHD symptoms [122]. Physical health challenges frequently manifest as chronic gastrointestinal disturbances [122]. The etiology of ASDs is multifaceted, involving a combination of genetic and environmental factors, such as synaptic dysfunction and plasticity across various neurotransmitter systems, transcription regulation and chromatin remodeling, protein translation and modification, neuroimmune regulation, and mitochondrial function [107].

#### 7.6.2 Metabolic Abnormalities in ASDs

Research has identified various metabolic abnormalities associated with ASDs. One of the most common metabolic abnormalities in ASDs is dysregulation of energy metabolism, particularly mitochondrial dysfunction [106, 123]. The prevalence of mitochondrial diseases in individuals with ASD is approximately 5.0%, which is 500 times higher than in the general population [107]. Nearly one‐third of children with ASD exhibit elevated plasma lactate levels and/or increased lactate‐to‐pyruvate ratios. Furthermore, other mitochondrial biomarkers, such as pyruvate, carnitine, and coenzyme Q_10_, show significant differences between individuals with ASD and controls [106]. Several genes involved in mitochondrial function are recognized as risk factors for ASD. For example, *SLC25A12*, which encodes the primary form of the mitochondrial aspartate/glutamate carrier [124]. These carriers play essential roles in mitochondrial functions, including respiratory control, calcium signaling, antioxidant defense, and mitigating glutamate‐mediated excitotoxicity [125]. Another notable gene, *TMLHE* (trimethyllysine hydroxylase ε), encodes the first enzyme in carnitine biosynthesis and has also been linked to ASDs [126]. Carnitine is crucial for transporting long‐chain fatty acids into mitochondria and maintaining normal mitochondrial function [127]. The gene encoding the mitochondrial inner membrane protease‐like protein IMMP2L may also contribute to ASD susceptibility [128]. Additionally, Mitochondrial dysfunction is believed to directly impair synaptic function, which could play a central role in the neurodevelopmental deficits observed in individuals with ASD [129].

Amino acid metabolism, for example, is frequently reported in children with ASD. An imbalance in the glutamate and GABA cycle, which is crucial for regulating excitatory and inhibitory neuronal signals, has been proposed to underpin the behavioral phenotypes associated with ASDs; however, findings regarding the direction and magnitude of these changes remain inconsistent [130]. Many studies have reported elevated blood glutamate levels in individuals with ASDs [131, 132, 133], which correlate with the severity of symptoms. Moreover, proton magnetic resonance spectroscopy has demonstrated higher brain glutamate concentrations, further supporting the hypothesis of excitatory/inhibitory imbalance [134]. These findings suggest that blood glutamate levels might serve as a potential biomarker for the early detection and stratification of ASD. Increased excitation or decreased inhibition might contribute to neuronal hyperactivity and disrupt the development of neural circuits, thereby affecting social and cognitive functions. This notion is supported by the high prevalence of epilepsy among ASD individuals [135]. However, contrasting evidence indicates that in certain brain regions, such as the striatum, reduced glutamate levels correlate with more severe social symptoms [136], while elevated GABAergic activity has been observed in specific animal models [130, 137]. Furthermore, delayed GABA system development may impact language learning, sensory processing, and emotional regulation [138]. In addition to these neurotransmitter imbalances, emerging research highlights that abnormal metabolism of other amino acids may influence synaptic plasticity and neuronal connectivity. Clinical cases involving functional mutations in branched‐chain α‐keto acid dehydrogenase kinase (BDK), linked to unrestricted BCAA oxidation, have been associated with ASDs and epilepsy. Animal models suggest dietary supplementation can improve outcomes in these cases [65, 139]. Impaired BCAA transport at the BBB has also been implicated in ASDs [140], and dysregulation of other amino acids may hold diagnostic significance [141].

Lipid metabolism abnormalities, particularly concerning cholesterol and essential fatty acids, also have been observed in ASDs. Both low and high cholesterol levels can affect neuronal membrane fluidity and signaling [142]. Additionally, reduced levels of essential fatty acids, particularly ARA and DHA, are common in ASD patients and may adversely affect neuronal growth and synaptic plasticity [10]. Furthermore, Individuals with ASD frequently exhibit elevated oxidative stress and compromised antioxidant defense systems [106]. These metabolic disturbances further exacerbate the neurological and behavioral challenges faced by individuals with ASD. Notably, dysregulation of energy metabolism, particularly mitochondrial dysfunction, is prevalent in ASD populations [106]. The prevalence of mitochondrial diseases among individuals with ASD is approximately 5%, significantly higher than the general population's rate of around 0.01% [107]. Elevated plasma lactate and altered levels of other mitochondrial biomarkers (such as pyruvate, carnitine, and coenzyme Q_10_) have been documented, suggesting a direct relationship between mitochondrial dysfunction and synaptic impairment in ASD patients [106].

## 8 Therapeutic Interventions

Addressing metabolic abnormalities through dietary and pharmacological interventions offers promising therapeutic avenues for individuals with ASD. Some studies suggest that supplementation with essential fatty acids, such as DHA and EPA, may support neurodevelopment and cognitive function, although additional research is required to confirm these effects [143]. The neuroprotective and anticonvulsant properties of the KD have shown particular promise in individuals with ASD, especially those with comorbid epilepsy [107]. Additionally, targeting neurotransmitter imbalances with GABA modulators like baclofen, or supplementing with branched‐chain amino acids (BCAAs), may help alleviate core or associated ASD symptoms [138, 139]. Given the role of gut dysbiosis in ASD, interventions such as FMT and supplementation with probiotics or prebiotics have demonstrated potential in improving gastrointestinal health and behavioral symptoms in ASD [119, 120]. Other nutritional supplements—including carnitine, vitamins, folinic acid, sulforaphane, and antioxidants such as coenzyme Q10, N‐acetylcysteine, and glutathione—have been studied for their ability to enhance mitochondrial function, reduce oxidative stress, and support neurodevelopment in ASD [106, 129]. For example, a randomized double‐blind study demonstrated that oral folinic acid can effectively and safely improve communication and behavioral symptoms in individuals with ASD [144]. Recent findings emphasize the neuroprotective and anti‐inflammatory effects of sulforaphane, a bioactive compound found in cruciferous vegetables like broccoli, which has been shown to significantly reduce irritability and hyperactivity symptoms in individuals with ASD in both preclinical and clinical studies [145, 146]. Antioxidants, by mitigating oxidative stress at the cellular level, may offer additional therapeutic benefits for managing ASD‐related pathophysiology [129]. Nevertheless, further well‐controlled studies are essential to confirm the efficacy, safety, and optimal application of these interventions in clinical settings.

## 9 Conclusions

The intricate interplay between neurodevelopment and metabolism underscores the importance of maintaining metabolic homeostasis for optimal brain function. This review highlights the pivotal roles of glucose, lipid, and amino acid metabolism, along with gut microbiome balance, mitochondrial dynamics, and key signaling pathways, in supporting the processes that drive neuronal growth, differentiation, and synaptic plasticity. Disruptions in these pathways are closely linked to the onset and progression of neurodevelopmental disorders, emphasizing the need for early detection and targeted interventions. Interventions aimed at restoring metabolic balance, including dietary modifications, microbial‐based therapies, pharmacological treatments, and innovative approaches like ketogenic diets and mTOR modulation, hold significant potential for improving neurodevelopmental outcomes and mitigating the impact of these neurodevelopmental disorders. Future research focusing on integrating genetic, environmental, and metabolic insights will enhance our understanding of neurodevelopmental processes. By advancing diagnostic tools and therapeutic strategies, we can pave the way for improving the quality of life for individuals affected by neurodevelopmental disorders, ultimately fostering healthier brain development and function.

## Author Contributions

L.Z. and Y.H. conceptualized the project and provided critical review and edits to the manuscript. Y.H. and L.Z. searched the literature and wrote the manuscript. Y.H., K.X., and K.Y. drew the illustration imaging. N.W. provided critical review and edits to the manuscript. All authors read and approved the final manuscript.

## Conflicts of Interest

The authors declare no conflicts of interest.

## Data Availability

No data was used for the research described in the article.
